# Towards the Measurement of Sea-Ice Thickness Using a Time-Domain Inductive Measurement System

**DOI:** 10.3390/s25020510

**Published:** 2025-01-16

**Authors:** Danny Hills, Becan Lawless, Rauan Khangerey, Jeremy Wilkinson, Liam A. Marsh

**Affiliations:** 1 Department of Electronic and Electrical Engineering, University of Manchester, Manchester M13 9PL, UK; becan.lawless@manchester.ac.uk (B.L.); rauan.khangerey@manchester.ac.uk (R.K.); 2 British Antarctic Survey, High Cross, Madingley Road, Cambridge CB3 0ET, UK; jpw28@bas.ac.uk

**Keywords:** electromagnetic sensing, time-domain metal detection, sea-ice thickness, broadband electromagnetic induction, EMI

## Abstract

Frequency-domain electromagnetic induction (EMI) is routinely used to detect the presence of seawater due to the inherent electrical conductivity of the seawater. This approach is used to infer sea-ice thickness (SIT). A time-domain EMI sensor is presented, which demonstrates the potential for correlating the spectroscopic properties of the received signal with the distance to the sea surface. This is a novel approach to SIT measurement, which differs from existing methods in that it uses measurements from 10 kHz to 93 kHz rather than a single frequency. The sensor was tested at a tidal pool containing seawater and measured to have a conductivity of 57.3 mS/cm. Measurements were performed at a range of heights between 0.2 m and 1.9 m above the sea surface and for inclinations from 0° to 45°. These measurements were correlated with Finite Element Modeling (FEM) simulations performed in COMSOL. The measured and simulated datasets are presented along with a proposed form of post-processing, which establishes a correlation between the distance to the sea surface and the measured EMI response. This forms a proxy measurement for the absolute distance from the EMI sensor to the sea surface. Because the air gap between the sensor and the seawater is indicative of the properties of sea ice, this study demonstrates a novel approach to non-destructive measurement of sea-ice thickness. The measurements show that this distance to the sea surface can be estimated to within approximately 10% of the known value from 0.2–1.5 m and 15% from 1.5 to 1.9 m.

## 1. Introduction

**Figure 1:**
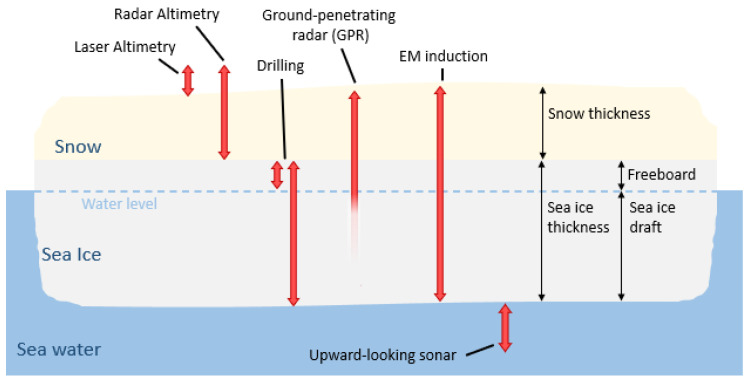
Comparison of methods used to measure sea-ice thickness.

The World Meteorological Organization (WMO) defines sea-ice thickness (SIT) as an Essential Climate Variable [1]. For almost 50 years, the area and extent of sea ice in the polar regions have been continuously monitored from a series of satellite-mounted passive microwave sensors [2]. For climate change studies, it is advisable to consider both the area of coverage and SIT together in order to determine changes in sea-ice volume. However, it has been particularly difficult to monitor SIT directly from space. Satellite altimetry, such as that from CryoSat-2 [3], can measure the height of sea ice above known sea level (known as freeboard, see Figure 1) and analytically determine SIT using several assumptions, such as hydrostatic equilibrium and snow depth as well as the density of the sea ice, snow, and ocean. Uncertainty in any of these assumptions can lead to erroneous SIT measurements, and additional modeling to account for snow layers is required [4]. There is, therefore, still a fundamental requirement for in situ measurement of SIT for calibration and validation of airborne and satellite data [5]. The traditional method for validation is to drill a hole through the sea ice via an auger and take a physical measurement using a T-anchor [5,6]. While this is a very accurate measurement, it suffers from the disadvantages of being time-consuming and destructive. Consequently, the approach offers poor spatial resolution, coverage, and repeatability.

Other, less destructive methods suffer from similar difficulties. Upwards-looking sonar, which uses acoustic waves to sound ice from the bottom, returns a measurement on the ice draft, the part of the ice underneath the waterline. As with freeboard from satellite altimetry, this requires assuming hydrostatic equilibrium and can have varying resolution depending on the temperature and type of ice [7], and is prone to errors when the ice bottom surface is not flat [8]. Buoys, particularly ice-mass-balance buoys, can have an excellent resolution of thickness paired with long-term measurement but require large numbers for spatio-temporal area coverage [9]. Ground-penetrating radar (GPR) has been shown to be effective in determining the thickness of freshwater ice. Though it struggles to penetrate sea ice as losses increase due to the existence of pockets of brine within the sea ice; this is particularly true for first-year ice [10].

Electromagnetic induction (EMI) sensors have been used to determine SIT for several decades. However, knowledge of the snow depth at the EMI measurement site is needed. This approach exploits the inherent conductivity of seawater, which is primarily a property of its salinity [11]. A time-varying current is applied to a coil of wire; this generates a primary magnetic field in the space surrounding the coil. This field leads to the induction of eddy currents in the seawater. These currents circulate through the conductive medium, therefore producing a secondary magnetic field. The resulting interaction between the primary and secondary fields can be measured by detecting changes in the magnitude and phase of the induced voltage of a nearby coil of wire. The response of EMI systems drops sharply as measurable targets become more distant; this is due to the characteristic decay of magnetic field strength at a distance. Consequently, it is possible to correlate the magnitude of the overall response of an EMI sensor and the overall from the target (i.e., sensor distance from the sea surface). Sea ice has an average conductivity of 0.17 to 0.894 mS/cm depending on environmental factors and axis of measurement; sea water is, on average, approximately 24 to 27 mS/cm in the Arctic [12]. As the conductivity of the ice is negligible compared with the seawater, this measurement assumes no contribution from the sea ice. Consequently, trials and calibration can be undertaken without the physical presence of ice or snow; in such cases, an air gap can be used to represent the sea-ice layer.

Perhaps the most commonly used system for measurements from the surface of the ice is the Geonics EM31 Ground Conductivity Meter, which was developed in 1976 and later updated in 1995 with a Mark-2 iteration of the hardware. There are several variants of this sensor, though all variants of the EM31 operate using a continuous 9.8 kHz frequency signal with transmit and receive coils placed at the ends of a plastic tube. In the original design coils are 3.66 m apart, and are 2.0 m apart in the short “SH” variant. The numerous variants of the system have been extensively deployed across many polar campaigns [13,14,15,16,17,18]. The system has been proven to be a useful tool for estimating SIT; however, it suffers from its design as a stand-alone sensor system that was not intended for sea-ice measurement. It is unaware of its global position, orientation, and height from the snow/ice surface. It can also be susceptible to vibration/breakage caused by moving it over the rough sea-ice surface. Each of these properties adds to the uncertainty of measurement and must be either assumed or separately measured with different sensors, which impact the accuracy of the data gathered, particularly in the case of orientation [19]. Additional evaluation of the system found that it is slow to react to changes in thickness and generally struggles with penetration depth for thicker multi-year ice [20]. Moreover, given the age of the system, it is envisaged that improvements can be made with SNR with digital signal processing, and a new generation of low-noise amplifiers.

The system used in this paper builds on the work conducted by Wilson et al. and employs a pulse induction (PI) inductive measurement system, which is specifically tailored for deployment in polar environments [21]. Previous generations of this detector have been deployed in Arctic and Antarctic environments [22,23]. PI methods can be more robust to vibrations in the coil than multi-coil, frequency-domain detection systems. This allows for application to a wide range of use cases, such as being towed over the sea ice on a sled. Also, as a result of being inherently broadband, the approach allows for more innovative post-processing techniques than frequency-domain systems, meaning development for greater flexibility in different situations is possible. This is because broadband measurement systems gather considerably more data about the target material when compared to single-frequency systems such as the EM31.

The work presented in this paper is believed to be the first reported instance of testing a purpose-built PI-based EMI sensor for the application of multi-frequency SIT measurement.

## 2. Description of Sensor System

### 2.1. Coil Geometry

**Figure 2:**
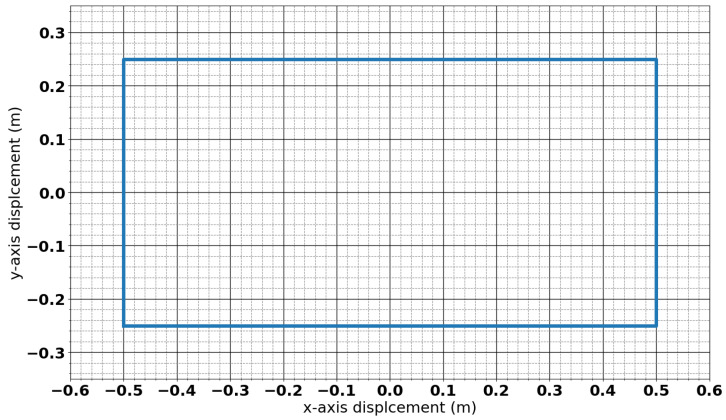
Coil geometry.

The detector system uses a single rectangular coil that measures 1 m × 0.5 m and consists of 15 turns of PVC insulated wire. The coil geometry is shown in Figure 2. The coil is embedded into a 0.9 m × 1.2 m panel of High-Molecular-Weight Polyethylene (HMWPE) and is held in place with epoxy resin. This reduces the chance of the coil deforming, which can add significant amounts of noise to measurements. This geometry and construction have been previously reported in [21] and has previously been tested in polar trials as reported in [22,23].

**Figure 3:**
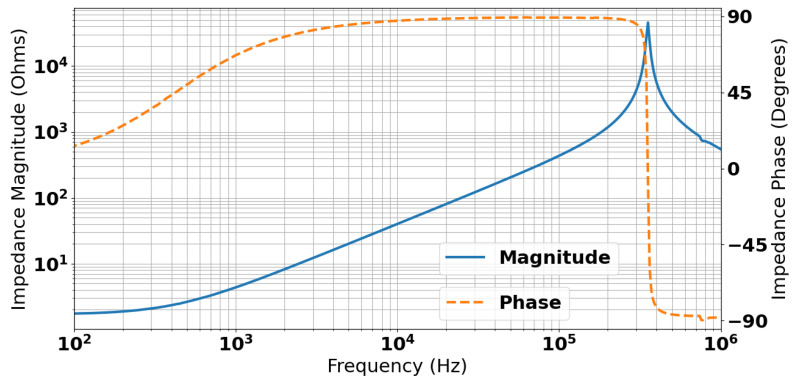
Measured coil impedance.

The impedance of the coil was measured using a Solartron 1260A impedance analyzer (Solartron Analytical, Farnborough, UK); this is shown in Figure 3. The figure shows that the coil has an inductive region between approximately 500 Hz and 200 kHz. Resonance of the coil occurs at approximately 350 kHz.

### 2.2. System Architecture

**Figure 4:**
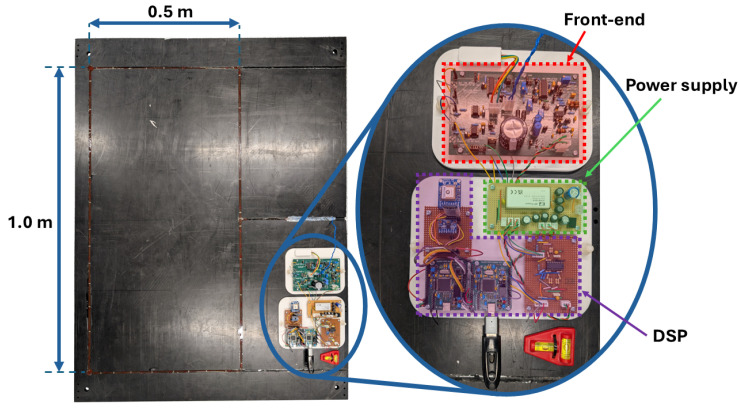
Image of coil panel and associated electronics.

**Figure 5:**
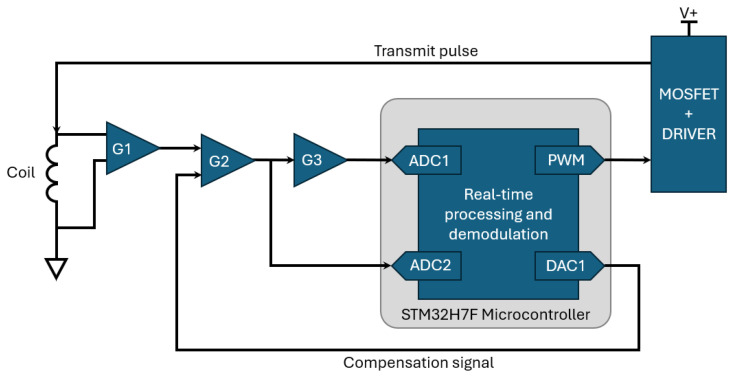
Architecture of EMI sensor.

As shown in Figure 4, the EMI electronics consists of three major components—microcontroller-based DSP hardware, front-end electronics for interfacing the microcontroller with the coil, and low-noise power supply circuitry. The front-end electronics are a modified implementation of the system reported in [21]. The system is composed of a transmit stage, which consists of a MOSFET and associated electronics for driving high current pulses through the coil, and a receive stage, which implements four gain stages. These gain stages include an instrumentation amplifier (G1 ≈ 16.36), a summing amplifier (G2 ≈ 1.94), a subsequent gain stage (G3 ≈ −10.87), and a final gain stage required to buffer and offset the input signal to the microcontroller (G4 ≈ 0.103 gain with a +1.65 V offset). An upgraded MOSFET has been added to the previously reported electronics. This enables faster switching transitions and significantly improves thermal performance. Other alterations include the replacement of the digital signal processing electronics, which is now performed on an STM32H723VGT6 microcontroller (STMicroelectronics, Geneva, Switzerland) [24]. The architecture of the system is shown in Figure 5; this excludes any buffering and offsetting stages required for interfacing with the microcontroller.

### 2.3. Operating Principles

The system generates rectangular 100 μs pulses at a frequency of 1 kHz. The pulses have a magnitude of 15 V and generate approximately 1.5 A current flow within the coil. A magnetic field is produced by the rapid current change at the falling edge of each pulse. The same coil is used to measure voltages that have been induced due to circulating eddy currents from nearby conductive or magnetic targets, in this case, the seawater. After each pulse’s falling edge, the coil voltage is sampled for 205 μs at a sample rate of 5 MHz. This measurement window has been chosen because it corresponds to 1024 samples per pulse cycle; this allows a radix-2 FFT to be performed on each dataset, therefore reducing the computational needs of the microcontroller.

**Figure 6:**
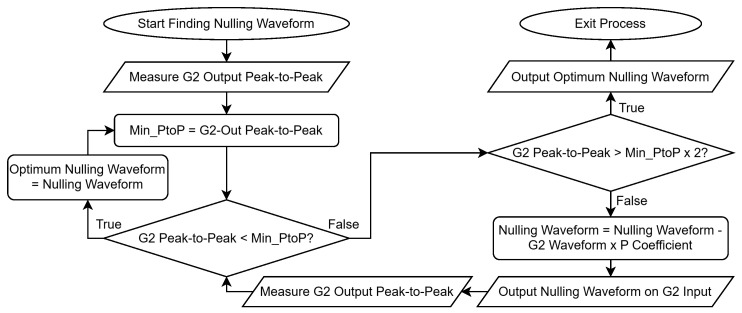
Flow chart showing the compensation waveform generation process.

Under background conditions (i.e., in the absence of measurable targets), the response measured at the output of G3 (Figure 5) shows large regions of saturation. This is due to the decaying primary field, and the components of the oscillations within the coil that could not be removed by hardware; these are unwanted signals which do not provide any useful information about target responses. To overcome these unwanted signals, this system implements a dynamic compensation process that can remove the background signal, therefore preventing saturation. By doing so this allows the majority of the dynamic range of the receive electronics to be used to measure the now isolated secondary voltages received from targets, which in this case is the seawater. The system uses a closed-loop controller to measure the output of G2 (Figure 5) and subsequently generate and store an appropriate waveform to self-null any background signals. Figure 6 shows the process of generating a compensation waveform for continuous injection. The flowchart shows a simplification of the process of subtracting the G2 Waveform × P coefficient, though, in practice, this is undertaken in the frequency domain. This, therefore, requires a live FFT of the signal, with components subsequently phase shifted by 180°. Additional calibration is also performed to ensure phase cohesion, which incorporates the system response of the closed-loop container; the coefficients for this are pre-computed and stored locally.

**Figure 7:**
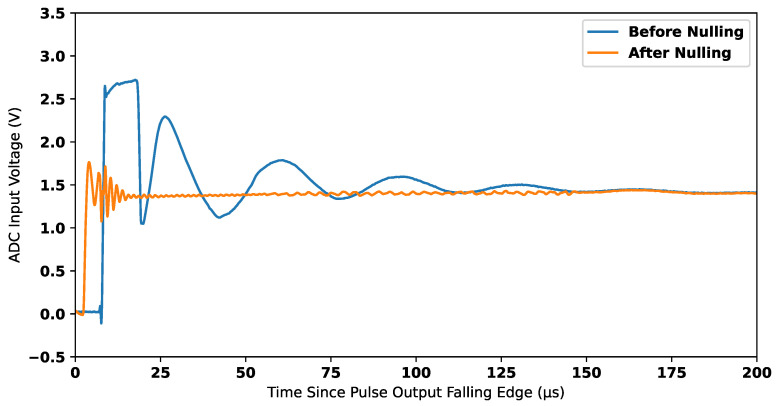
Signal nulling after gain stage 2 (G2).

**Figure 8:**
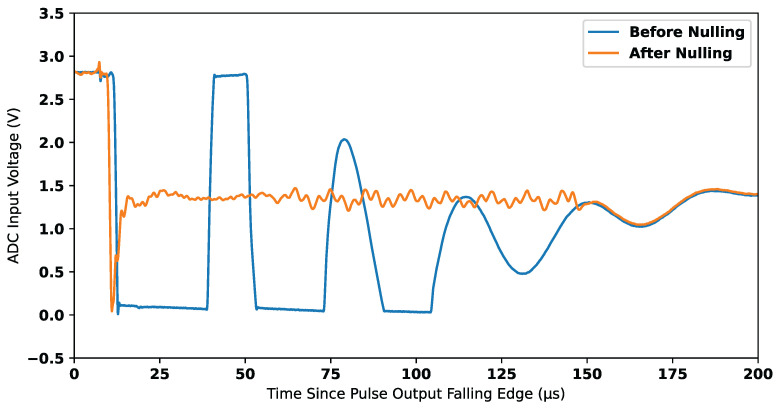
Signal nulling after gain stage 3 (G3).

When a stable compensation signal is achieved, the amplifier G3 shows a near-zero output in the absence of a measurable target. When the system is subsequently moved into the vicinity of a measurable target, this misbalances the system and leads to a non-zero voltage at the amplifier G2; this is further amplified by G3. Figure 7 shows the output of amplifier G2 in cases with and without a compensation signal being used. This demonstrates the viability of the process to reject background signals and consequently extend the dynamic range. By reducing the dynamic range requirements of G2, amplifier G3 is no longer saturated. Figure 8 shows the output of G3 in cases with and without a compensation signal being used.

## 3. Field Trials

### 3.1. Location

**Figure 9:**
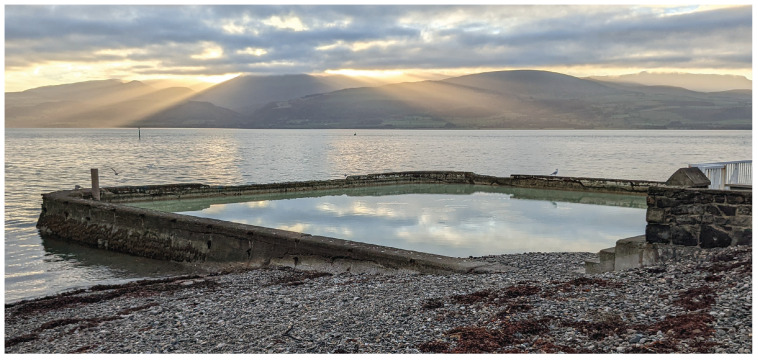
Image of test site taken on the day of testing.

The field trials were conducted in the town of Beaumaris, Anglesey, UK. A man-made tidal pool that naturally fills at regular intervals was selected due to having a fixed depth and calm water conditions throughout the experiment. This would not be the case for the open sea, where tides, wind, and waves would cause varied depths and operating conditions and, therefore, increase experimental uncertainties. The pool is located at 53°15′55.5192″ N, 4°5′9.78″ W, and is shown in Figure 9. Samples of the seawater were taken from the pool and the adjacent ocean; these were measured with electrical conductivities of 57.3 mS/cm and 58.8 mS/cm, respectively. Therefore, the water within the pool is considered to be representative of seawater, which has a mean oceanic salinity of 52.6 ms/cm [25]. This value is slightly above the mean electrical conductivity of seawater in polar regions, as stated previously, as 24 to 27 mS/cmbe. Though there is a small difference in these values, it is sufficient to evaluate the performance of the detection system as they are of the same order of magnitude. This allows the ability to establish underlying correlation with simulation and to propose algorithmic approaches to quantifying absolute lift-off (distance between the sea surface and sensor system) values as a proxy for SIT. The conditions on the day of testing were clear, with an air temperature of 12 °C, and a wind speed of 9 km/h.

**Figure 10:**
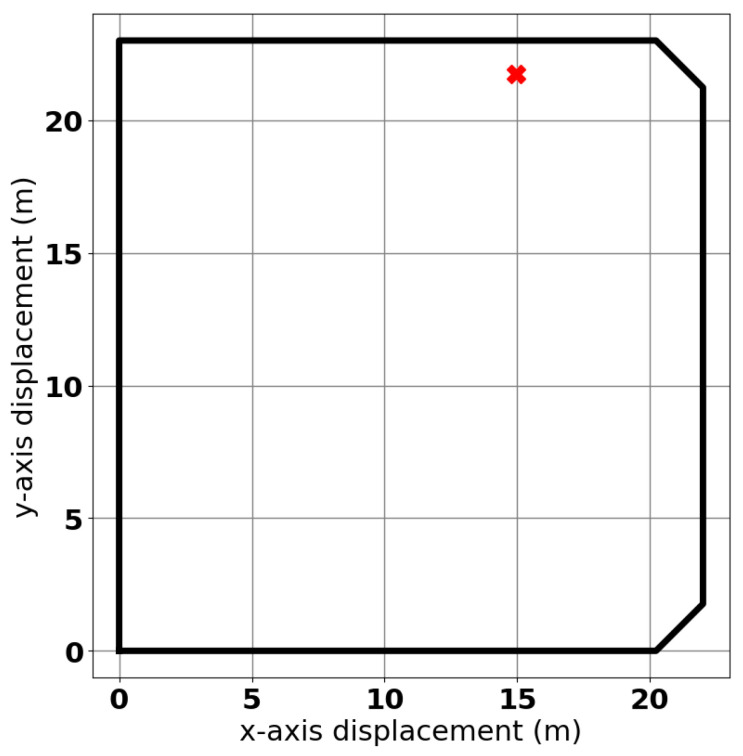
Approximate location of the experiment within the tidal pool.

The dimensions of the pool were measured to be 22 m × 23 m. There was some minor variation in depth across the volume of the pool, but a test area was selected with a measured constant depth of 1.7 m. A survey of the pool was conducted to check for large ferritic clutter using magnets, and no such contamination was found. Small perturbations in sensor placement were made to ensure that no significant metallic contamination was present, and no such targets were found. A section of the pool 1 m from its northernmost retaining wall was therefore deemed to be a suitable testing environment. The approximate location within the pool is shown in Figure 10.

### 3.2. Experimental Methodology

**Figure 11:**
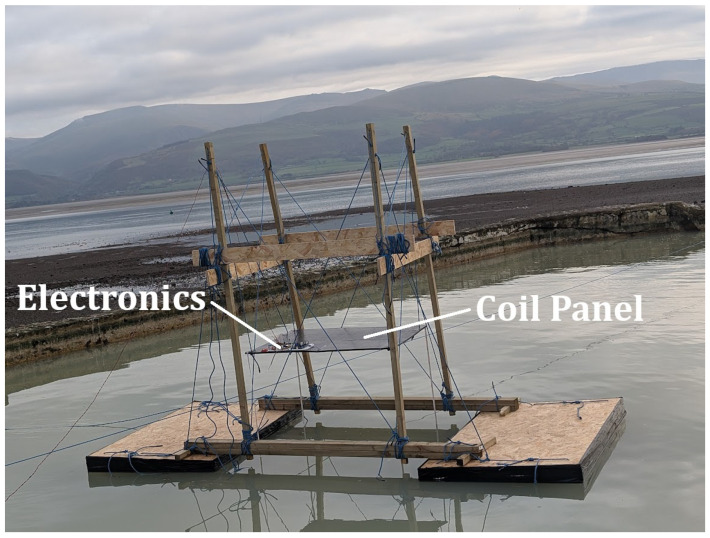
Arrangement of the coil panel and metal-free rig to control lift-off.

A bespoke, fully non-metallic test rig was designed and constructed to carry out tests over water. This consisted of floating the sensor array on two pontoons made from oriented strand board (OSB) wooden sheets and expanded polystyrene foam. This foam was sealed at the edges with water-resistant duct tape to mitigate water absorption by the foam. A spacing of 1.7 m was maintained between the pontoons, providing a sufficiently unimpeded view of the level water surface. A wooden structure was constructed on top of the pontoons, which allowed the panel containing the coil and sensor electronics to be raised and lowered from approximately 0.15 m to 2.1 m above the water level. Polypropylene rope was used for securing the various wooden structural elements, as well as to ensure the stability of the vertical posts. This arrangement allowed for the panel to be manually oriented in two axes. This was achieved by separately tensioning four ropes at each corner of the panel. This arrangement is shown in Figure 11. The rig and surrounding free space constitute a metal-free zone in which the seawater is the only conductive material.

Several measures were implemented to characterize the position and orientation of the panel with respect to the water. The panel featured a weighted, submersible cloth measurement tape in each corner. The tape measures were all zeroed to the top surface of the panel, which had a thickness of 15 mm. This allowed each corner’s distance to the water to be set individually via appropriately tensioned rope, ensuring a uniform lift-off from the water.

Multiple methods were made to estimate measurement uncertainty. These included video recordings of the tape measures at the waterline showing perturbations on the scale of ±3 mm due to flexing of the system and water ripples, and the calibration of the cloth tape measures weighted vs unweighted against a metal rule showing maximum extensibility of 1 mm over 1 m. While every effort was made to set each lift-off as accurately as possible, we recognize a potential limitation of viewing the tape measures from a distance and from small variations to be introduced while tying off.

A secondary measurement system was attached to the coil panel. This included an accelerometer, which used a 6-axis inertial measurement unit (IMU) capable of characterizing the roll, pitch, and yaw of the panel. A GPS was also present to ensure real-time synchronization and measurement of the sensor location. This system transmitted data wirelessly and in real-time to a nearby computer. Consequently, it was possible to confirm the panel orientation prior to each measurement being taken. The data were synchronized with the EMI sensor. This allowed for orientation in the panel to be investigated as a source of noise, and to support the estimated measurement uncertainty in the experiment.

Accounting for the potential uncertainties of the tape measurements and the inclination variations of the sensor panel in operation, the overall uncertainty in the water-to-panel lift-off is estimated to be within ±10 mm.

A total of four measurement sets were collected for lift-off measurements. These were structured as two datasets from 1.9 m down to 0.2 m in 10 cm intervals and two further datasets from 1.0 m down to 0.2 m, also in 10 cm intervals. ’Experiment 1’ and ’Experiment 2’ refer to the 1.9 to 0.2 m variations, and ’Experiment 3’ and ’Experiment 4’ refer to the 1.0 to 0.2 m variations. This allowed for four datasets in the region of 0.2 to 1.0 m and two datasets in the range of 1.0 m to 1.9 m.

A further experiment was undertaken to investigate the extent to which the orientation of the sensor introduces error in lift-off estimation. A measurement set was recorded where the sensor was fixed at a height of 1.0 m and oriented from 0° to 45°. The results of this experiment and associated discussion are provided as a separate study in Section 5.3.

A total of 20 s of data were captured for each measurement, i.e., at each lift-off and orientation the panel was set to; this corresponds to approximately 11,840 raw EMI measurements. The secondary measurement system recorded 200 orientation values during each 20 s interval. The quantity of data recorded across these two systems provides a clear indication of the electrical stability of the EMI sensor and the mechanical stability of the coil. These are presented in the form of the standard deviation of datasets in Section 5.

## 4. Modeling and Simulation

Simulations were performed to validate the experimental outcomes from the in situ experiments (Section 3) and to provide a baseline for the use of the simulated models as a vehicle for future design modifications. Simulations were conducted in COMSOL version 6.1 using its ‘Magnetic Fields Interface’. COMSOL is a multi-physics software package that uses Finite Element Modeling (FEM) to simulate physics problems [26].

The simulated coil was excited with measured current data from the true system electronics input to the time-domain study using a COMSOL (piecewise cubic) interpolation function. These data were created from measured voltage waveforms across a shunt resistor in series with the coil, with a maximum current of 1.5 A.

COMSOL can calculate the eddy currents induced in the seawater as a result of the coil excitation current. The coil and seawater can be modelled as a mutual inductance using Equation (1) [27]. This shows that there is a linear correlation between the rate of change in current in the seawater ($\frac{dI_{a}}{dt}$) and the corresponding voltage induced in the coil ($V_{b}$). Though COMSOL is capable of simulating $V_{b}$, this requires an extra simulation step to relate this with $\frac{dI_{a}}{dt}$. The nature of FEM is such that each simulation step increases computational resource requirements and increases the model’s degrees of freedom, leading to further error in the simulation output [28]. By exploiting the linear relationship between $\frac{dI_{a}}{dt}$ and $V_{b}$, it is, therefore, possible to reduce simulation error and complexity by directly relating the trends in $\frac{dI_{a}}{dt}$ and $V_{b}$.

(1) $$V_{b}=-M\frac{dI_{a}}{dt}$$

**Figure 12:**
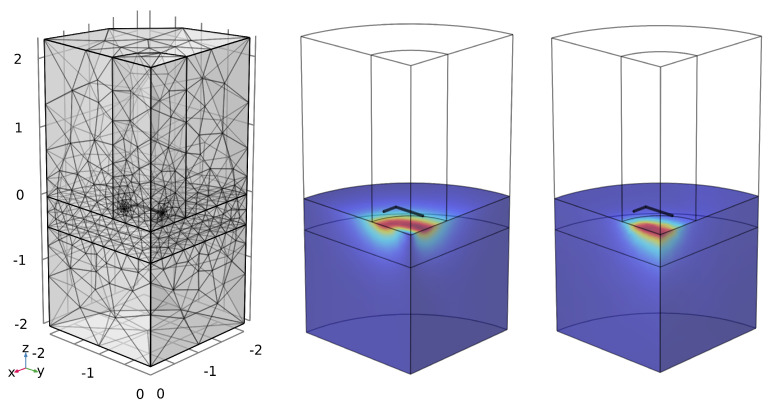
(**Left**) FEM Mesh for 3D Simulation, (**Center**) Current density norm for 0.2 m coil lift-off, (**Right**) Magnetic flux density norm for 0.2 m coil lift-off.

A 3D simulation is required as the rectangular geometry of the coil inherently changes its magnetic field, as compared to a cylindrical coil, and so an axisymmetric model would insufficiently describe the system. For the 3D simulation, a quarter model was used, taking advantage of the two planes of symmetry on the panel, with a magnetic insulation boundary condition for each symmetry plane, thus reducing the computation cost. This boundary condition enforces a mirror symmetry for current across the quartering planes, which can be used since the current at each will flow normally to the boundary surface [29]. As most of the excitation in the water will be near the surface due to the skin effects, it is desirable to keep the mesh in the water region below the coil as dense as possible. Skin depth is defined in a good conductor as the distance over which 86.5% of the power is lost by Equation (2) [30]. Figure 3 shows that the coil has an inductive region between approximately 500 Hz and 200 kHz. Based on values of $\mu =4\pi 10^{-7}\;{N/A}^{2}$ and σ=5.73 S/m, Equation (2) would give a skin depth of 9.26 m to 0.46 m with the majority of the energy from the system skewing towards the shallower depth value. A region of higher mesh density of 0.46 m was included at the surface of the water region, with a minimum mesh size of 0.153 m to provide at least 4 mesh nodes in the skin depth for the highest frequency of interest. The coil was represented as a homogenous multiturn coil with 15 turns each of cross-sectional area $2.2\times 10^{-7}$ m^2^. The 3D mesh can be seen in Figure 12. This figure also contains example plots of the current density and magnetic flux density, respectively, at time $1.15\times 10^{-4}$ s; a time immediately following the driven current drop-off in the sensing coil.

(2) $$\begin{array}{c} \delta_{s}=\frac{1}{\sqrt{\pi f\mu \sigma }} \end{array}$$

**Figure 13:**
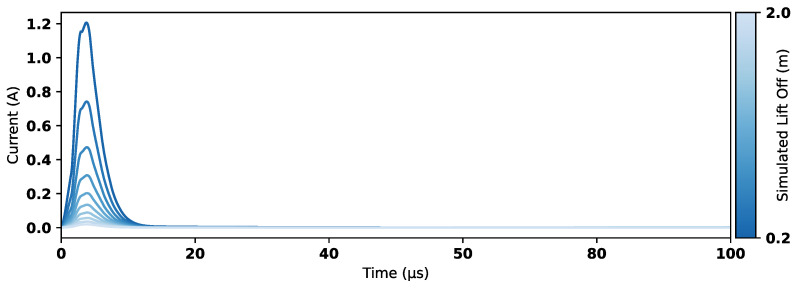
Example induced current integral in water for coil at 0.2 m lift-off in 3D model.

To keep the time steps sufficiently small within the period immediately following the current drop-off, the COMSOL implementation of a generalized alpha method [31] was used with free adaptive time stepping and a maximum timestep of $2.5\times 10^{-8}$ s. A parametric sweep was run for coil lift-off heights from 0.2 m to 2.0 m with 0.2 m increments. The simulation output consists of a set of induced eddy currents in the seawater as a function of sensor lift-off, as shown in Figure 13. These trends are compared with measured data in Section 5.

Table 1 contains a summary of the key parameters that went into simulating the 3D model of the sensing coil.

## 5. Results and Discussion

### 5.1. Field Trials

**Figure 14:**
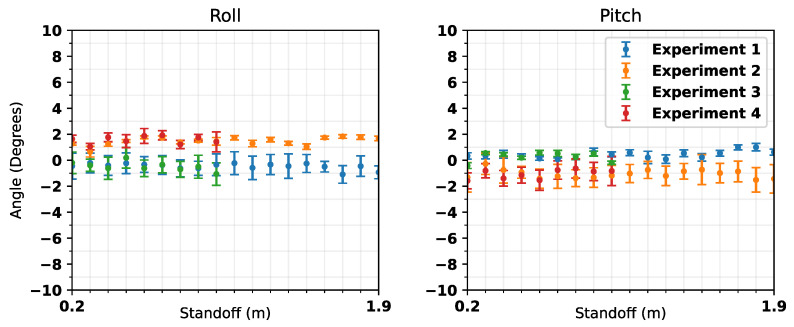
Variations in the roll and pitch of the panel during measurements. Error bars = ±2 standard deviations.

Figure 14 shows the recorded inclination of the panel with respect to both roll and pitch. The results show that the orientation of the panel did not change significantly while recording the measurements. The maximum deviation in roll and pitch does not typically vary by more than ±2°, though in most cases is well below this. Therefore, variation in orientation is not considered to have been significant, and the assumption is made that the panel can be considered to have been static while collecting data at each location.

**Figure 15:**
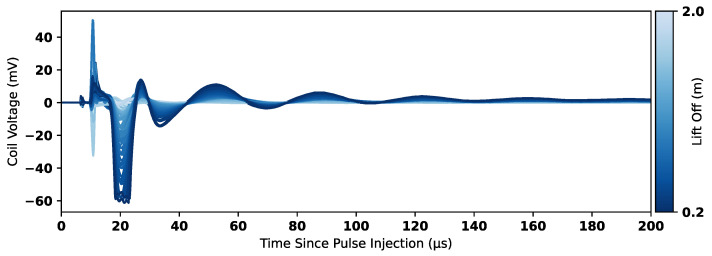
Measured coil voltage in reference to the 2.0 m measurements against time.

**Figure 16:**
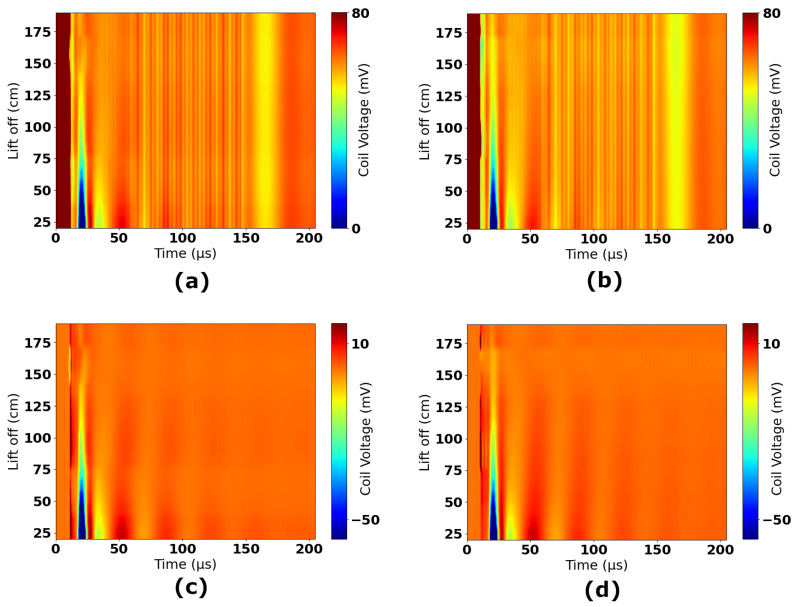
Time-domain plots of raw measurements (**a**) Experiment 1—raw waveforms, (**b**) Experiment 2—raw waveforms, (**c**) Experiment 1—with background subtraction, (**d**) Experiment 2—with background subtraction.

Figure 15 shows all experimental data collected in Experiments 1 to 4. The data have been scaled to account for gain stages and, therefore, correspond to the induced voltage in the coil. The reference 2.0 m time-domain response has been subtracted from all measurements to remove any resulting signal components that were not nulled in hardware by the injection of the compensation signal. Figure 16 shows the time-domain measurements separated by experiments; sub-figures (a) and (b) show the raw data for Experiments 1 and 2, respectively, and sub-figures (c) and (d) show the result of subtracting the 2.0 m reference. These figures show that the subtraction removes a significant amount of high-frequency information and DC offsets that are constant across all measurements. The resulting plots in Figure 16c,d, and in Figure 15 therefore focus on only the signal components that vary as a function of lift-off.

### 5.2. Post-Processing

**Figure 17:**
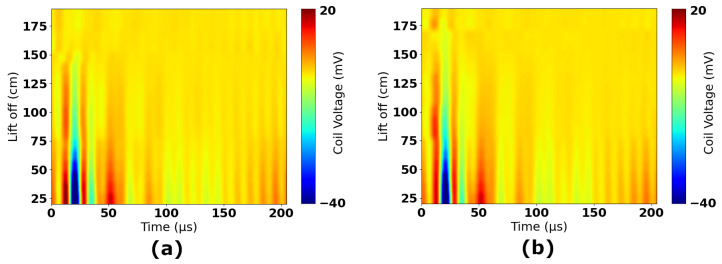
Time-domain plots of post-processed measurements (**a**) Experiment 1, (**b**) Experiment 2.

RMS values of the time-domain waveforms are indicative of the DC equivalent voltage; they are, therefore, correlative to the change in energy of the coil. RMS magnitude can be correlated with lift-off due to the fact that this change in energy is caused by eddy currents in the seawater. SNR can be improved by windowing time-domain waveforms and performing frequency-domain filtering. Through a process of iterative analysis, the optimal frequency range for observation was found to be 9762 Hz to 92,773 Hz. Figure 17 shows the result of band-limiting waveforms to this frequency range. Comparing Figure 17a,b with Figure 16c,d shows the impact of this filtering. Further gains in SNR can be made by windowing the RMS calculation. The optimal measurement window was found to be between 14.8 μs and 203.8 μs after the pulse’s falling edge. This improves lift-off measurements significantly, improving standard deviation by ≈11% for measurements below 1.0 m and ≈58% for measurements above 1.0 m.

**Figure 18:**
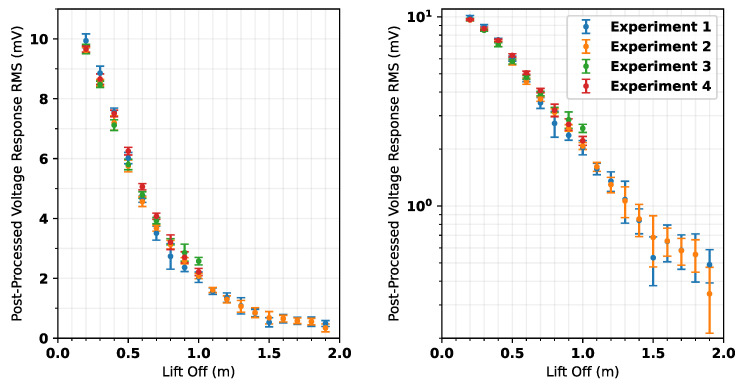
Post-processed measurement voltage RMS with lift-off (**left**) linear scale, (**right**) logarithmic scale. Error bars = ±2 standard deviations.

Figure 18 shows the mean RMS per lift-off as calculated for each experiment. The plots show that Experiments 3 and 4 exhibited a notable increase in RMS when compared with Experiments 1 and 2. This is thought to be because Experiments 3 and 4 utilize the same compensation waveforms as Experiments 1 and 2, respectively, whereas nulling was repeated at the beginning of Experiments 1 and 2. It is hypothesized to be caused by drift in the background signal, meaning that the active nulling is becoming less effective as time goes on. This is characteristic of nulling the system with respect to background signals present at the beginning of an experiment. Further characterization of the system is needed to mitigate this. This conclusion is supported by the observation that although Experiments 3 and 4 show differing mean values to Experiments 1 and 2, they exhibit very similar standard deviations.

The results show that repeatability drops between Experiments 1 and 2 over the range of 1.5 to 2 m. There are two heights where measurements vary significantly between each experiment (though standard deviations still overlap); these are 1.5 m and 1.9 m. There is no pattern to these anomalies, i.e., they are isolated discrepancies between datasets and not part of a clear trend. These differences are considered to be consequences of unintended deformation of the coil panel. After conducting Experiment 1, it was observed that the panel was experiencing significant stresses from the measurement rig when close to the highest achievable lift-offs. This is caused by increased tensioning of ropes as they reach their limits of travel. If this stress resulted in a distortion of the panel, it would have changed the characteristics of the received waveform. This can be seen in Figure 16c, where a significant trough is introduced at around 15 μs for lift-off values from 1.5 to 1.8 m. This also leads to a characteristic shifting of the waveforms in the time period <50 μs, which can be seen in Figure 16a,c, and also in Figure 17a. For Experiment 2, a slight change was made to the tensioning of the panel, though the location and orientation of the panel were kept the same between both experiments to allow for valid comparison. It can be seen that there is a significant reduction in the trough in Figure 16d. Similarly, there is no clear shifting of the waveform in Figure 16b,d. Therefore, it is likely to conclude that deformation is impacting the quality of the data gathered for these lift-offs. Figure 15 also clearly shows the impact of this; there is a trough at 15 μs, which is only present for colors relating to the 1.5 to 1.8 m heights. It is encouraging that despite this, there is still very good repeatability between the experiments, as shown in Figure 18. Both experiments show the same clear trend, which has been demonstrated to vary discernibly over the range of lift-offs used in this experiment.

Given the correlation between post-processed RMS voltage response and lift-off, it is possible to map these variables to one another. It was found that the simplest equation to map RMS to lift-off was created by first plotting the lift-off against RMS, with RMS being a logarithmic scale, then fitting a cubic polynomial. Equation (3) shows the resultant polynomial mapping lift-off (*d*) to post-processed RMS voltage (*x*).

(3) $$d=-0.501527\times \log{\left(x\right)}^{3}-3.92586\times \log{\left(x\right)}^{2}-11.2429\times \log\left(x\right)-10.6029$$

where

(4) $$x=\sqrt{\frac{1}{n}\sum_{t=t_{0}}^{T}V{\left[t\right]}^{2}}$$

In Equation (4), *V* refers to the sampled voltage at time *t*, $t_{0}=14.8$ μs, T=203.8 μs, and 0 μs corresponds to the falling edge of the injected pulse; *n* is a constant of 945, which represents the number of samples in each windowed waveform.

**Figure 19:**
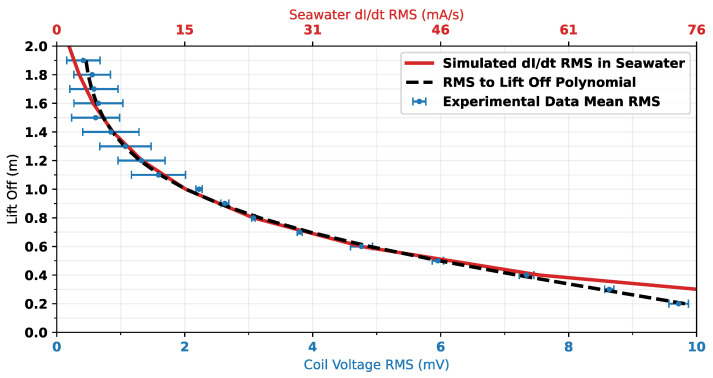
Comparison of measurement data, simulated data, and mapping polynomial shown in Equation (3); Error bars = ±2 standard deviations.

As RMS voltage is being used for correlation with lift-off, to compare this with simulation data for $\frac{dI}{dt}$, the RMS of $\frac{dI}{dt}$ has been calculated. Figure 19 shows the correlation between measurement data and simulated $\frac{dI}{dt}$ in the seawater. The mapping polynomial (Equation (3)) is also plotted for comparison. The figure shows that there is excellent agreement in the trends of measured RMS voltage and simulated $\frac{dI}{dt}$ RMS. The discrepancy between simulation and experimental data trends at lift-offs below 0.5 m is thought to be due to the saturation of the measurement waveform in this region. This saturation can be seen in Figure 15 at approximately t=20 μs.

**Figure 20:**
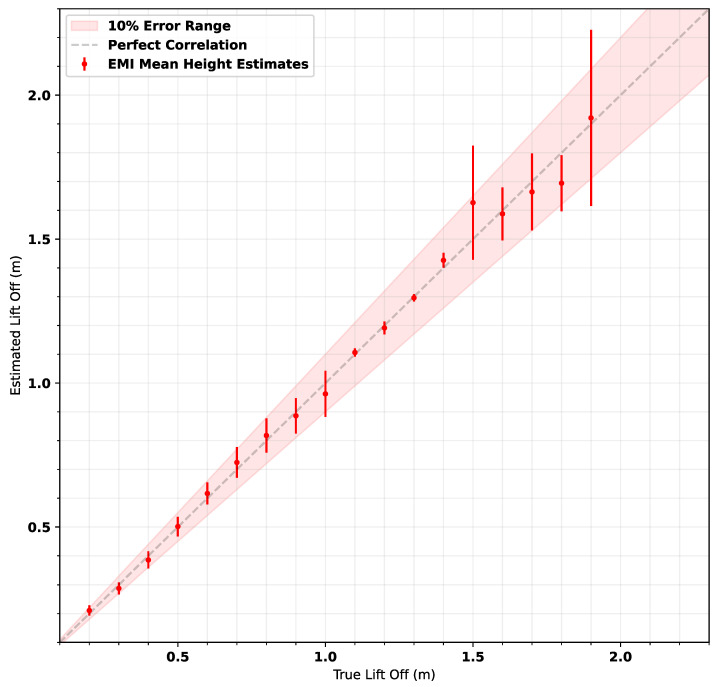
EMI lift-off estimates for all measurements taken on the field trial against the true lift-off of the measurement, with a perfect correlation line marked and 10% error region shown for comparison; Error bars = ±2 standard deviations.

Figure 20 shows the mean estimated lift-offs against true lift-offs for all 8320 measurements collected throughout the trial. The graph shows that when excluding the two anomalous lift-offs at 1.5 m and 1.9 m, estimates are within 10% of the true value.

### 5.3. Investigation of Sensor Orientation

For a practical deployment of a SIT measurement system, the system will likely encounter inclines as it traverses the sea-ice surface. This is problematic for existing EMI systems, which do not include technology for the measurement of orientation. Users are required to do all they can to ensure a constant orientation and accept any inaccuracy that accidental misalignment may cause.

**Figure 21:**
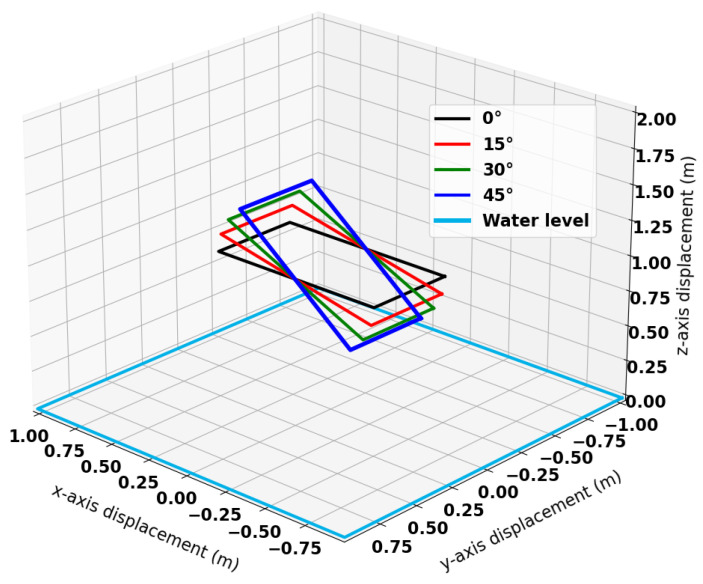
Orientations of the coil used for this investigation.

An additional experiment was conducted to quantify the impact of inclination on the sensor response. In this experiment, the sensor was fixed at 0° roll and pitch at a height of 1 m above the water. The system was nulled such that the response of the sensor was zeroed in this position and orientation. The sensor was then rotated about 15° offsets with respect to roll, as shown in Figure 21.

**Figure 22:**
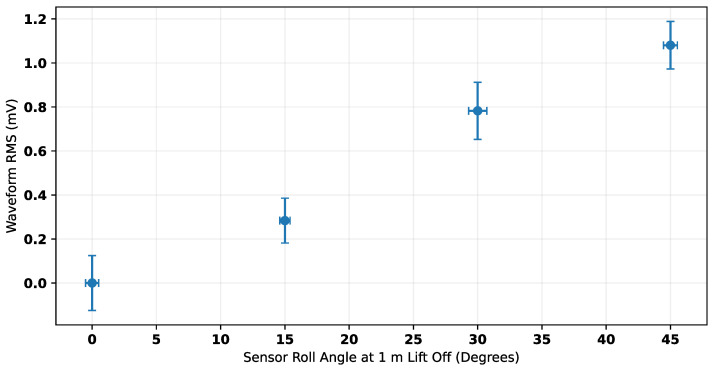
Expected RMS error introduced due to inclination. Error bars = ±2 standard deviations.

Response waveforms (as such in Figure 15) were referenced to the 0° waveform. The resultant waveforms could then be considered to be errors introduced by inclining the sensor. For comparison with the RMS trend used to estimate lift-off (Figure 18), the RMS of these waveforms is shown in Figure 22. The values in this plot can be considered representative of the additional expected error introduced in the RMS measurement of the response for each inclination tested. Table 2 shows the change in lift-off estimate introduced by inclination.

This highlights some very important considerations. First, it confirms that the impact of orientation on existing systems is likely to introduce significant errors in estimated thickness. However, it justifies a need to augment next-generation SIT measurement systems with auxiliary sensors that can measure the orientation of the sensor. Such sensors could either be used to provide feedback to operators on achieving a correct orientation or perhaps used as part of the algorithmic determination of the ice thickness when the sensor is known to be inclined.

## 6. Conclusions and Future Work

The results shown in this paper demonstrate the potential to correlate the absolute lift-off from the sea surface with measurements from a time-domain EMI system. Although the measurements have been post-processed, the algorithm can easily be implemented on the existing DSP hardware to enable real-time measurements. The trends in measurements have also been verified independently by means of a FEM simulation. This correlation shows that if the simulation is expanded to include secondary field voltage effects on the coil, it should be possible to include features from polar environments, e.g., snow, sea ice, and brine pockets. This could allow the prediction of the behavior of the system when exposed to these features. This can be used to accelerate the development of further prototypes and to enhance readiness for cold weather trials on sea ice, which are scheduled to commence in March 2026. The current generation of prototypes is only designed to take static measurements, whereas future generations will be sled-mounted and, therefore, capable of being dragged across the ice surface. There are, therefore, additional challenges to address in terms of mitigating the impact of vibration and investigating the impact of a reduced measurement integration time.

The sensor has shown the ability to repeatably and reliably quantify a range of lift-off values, which would enable the measurement of first-year ice, which has a maximum thickness of 2 m [32]. However, greater sensitivity is needed to be able to measure ridged ice, which is often thicker than 2 m. So, it is necessary to explore methods to enhance the sensitivity of the system. This could include optimization of the coil geometry, and varying the front-end electronics to improve SNR. This would encompass an attempt to further reduce the measurement uncertainty to be less than the current 10–15%.

There have been shortcomings identified in this study whereby significant errors in measurements occur close to the limit of maximum lift-off for the measurement rig. Although the system is still usable over this range of distances, there is a need to improve the robustness of the coil panel for future testing. However, it should be noted that the stresses that were being applied to the panel in this experiment are not representative of stresses that would be experienced during cold weather deployment, i.e., the panel would not be artificially suspended above the sea, but rather lying flat with lift-off determined by the thickness of the ice.

The results in this paper show a clear need to augment the proposed system with secondary measurement systems capable of quantifying the orientation and position of the sensor. There will be further work to incorporate this information in the algorithmic approach presented in this paper. Even with no further advancements in SNR, this approach could reduce the errors introduced by sensor misalignment. This has the potential to significantly improve the quality and robustness of in situ ice thickness measurements.

## Figures and Tables

**Table 1:** Key parameters for COMSOL 3D simulation.

Description	Value	Units
Seawater electrical conductivity	5.73	S/m
Seawater relative permeability	1	
Seawater relative permittivity	80	
Multiturn coil number of turns	15	
Wire cross-sectional area	$2.2\times 10^{-7}$	m^2^
Solver	MUMPS	
Time-dependent solver maximum timestep	$2.5\times 10^{-8}$	s
Time-dependent study user-defined relative tolerance	0.01	

**Table 2:** Lift-off estimate changes caused by different inclinations.

Sensor Inclination	Change in Lift-Off Estimate
15°	5.46 cm
30°	18.92 cm
45°	30.19 cm

## Data Availability

The original data presented in the study is openly available via Dropbox. Available online: https://tinyurl.com/5b2rxhe (accessed on 14 January 2025). The authors will maintain this dataset for a period of five years after the accepted publication date. The data presented in this study is also available on request from the corresponding authors.

## Abbreviations

The following abbreviations are used in this manuscript:

DSP	Digital signal processing
EMI	Electromagnetic induction
FEM	Finite element modeling
FFT	Fast Fourier transform
GPR	Ground-penetrating radar
GPS	Global positioning system
HMWPE	High-molecular-weight polyethylene
IMU	Inertial measurement unit
MOSFET	Metal-oxide semiconductor field-effect transistor
OSB	Oriented strand board
PI	Pulse induction
PVC	Polyvinyl chloride
SIT	Sea-ice thickness
SNR	Signal-to-noise ratio
WMO	World Meteorological Organization
